# Integrating a Numerical Taxonomic Method and Molecular Phylogeny for Species Delimitation of *Melampsora* Species (Melampsoraceae, Pucciniales) on Willows in China

**DOI:** 10.1371/journal.pone.0144883

**Published:** 2015-12-17

**Authors:** Peng Zhao, Qing-Hong Wang, Cheng-Ming Tian, Makoto Kakishima

**Affiliations:** 1 Graduate School of Life and Environmental Sciences, University of Tsukuba, Ibaraki, 305–8572, Japan; 2 State Key Laboratory of Heavy Oil Processing, Beijing Key Laboratory of Oil & Gas Pollution Control, China University of Petroleum, Beijing, 102249, China; 3 The Key Laboratory for Silviculture and Conservation of Ministry of Education, Beijing Forestry University, Beijing, 100083, China; 4 Faculty of Life and Environmental Sciences, University of Tsukuba, Ibaraki, 305–8572, Japan; 5 Engineering Research Center of Chinese Ministry of Education for Edible and Medicinal Fungi, Jilin Agricultural University, Changchun, Jilin Province, 130118, China; University of Szeged, HUNGARY

## Abstract

The species in genus *Melampsora* are the causal agents of leaf rust diseases on willows in natural habitats and plantations. However, the classification and recognition of species diversity are challenging because morphological characteristics are scant and morphological variation in *Melampsora* on willows has not been thoroughly evaluated. Thus, the taxonomy of *Melampsora* species on willows remains confused, especially in China where 31 species were reported based on either European or Japanese taxonomic systems. To clarify the species boundaries of *Melampsora* species on willows in China, we tested two approaches for species delimitation inferred from morphological and molecular variations. Morphological species boundaries were determined based on numerical taxonomic analyses of morphological characteristics in the uredinial and telial stages by cluster analysis and one-way analysis of variance. Phylogenetic species boundaries were delineated based on the generalized mixed Yule-coalescent (GMYC) model analysis of the sequences of the internal transcribed spacer (ITS1 and ITS2) regions including the 5.8S and D1/D2 regions of the large nuclear subunit of the ribosomal RNA gene. Numerical taxonomic analyses of 14 morphological characteristics recognized in the uredinial-telial stages revealed 22 morphological species, whereas the GMYC results recovered 29 phylogenetic species. In total, 17 morphological species were in concordance with the phylogenetic species and 5 morphological species were in concordance with 12 phylogenetic species. Both the morphological and molecular data supported 14 morphological characteristics, including 5 newly recognized characteristics and 9 traditionally emphasized characteristics, as effective for the differentiation of *Melampsora* species on willows in China. Based on the concordance and discordance of the two species delimitation approaches, we concluded that integrative taxonomy by using both morphological and molecular variations was an effective approach for delimitating *Melampsora* species on willows in China.

## Introduction

Currently, fast-growing woody crops are emerging as an attractive source of biomass. Among them, willows (especially shrubs) are one of the best candidates for the production of renewable energy and bioproducts [1, 2]. Moreover, willows are widely used for process such as phytoremediation, ornamentation and fiber production [3]. Leaf rust diseases caused by rust fungi from the genus *Melampsora* are some of the most widespread and common diseases that occur in natural habitats and plantations [4]. Diseases caused by *Melampsora* species have emerged as one of the most important factors limiting the development of willow cultivation. To date, approximately 90 species in the genus *Melampsora* have been reported worldwide, over 50 species of which have been reported as causal agents of leaf rust diseases on willows [5, 6]. These species were variously described in Asia, Australasia, Europe and North America, and were recorded with either heteroecious or autoecious life cycles [7–10].

*Melampsora* species on willows were recorded mainly as macrocyclic, with five different spore stages (spermagonium, aecium, uredinium, telium and basidium) were produced during the life cycle [11]. Different morphological characteristics were produced in these five spore stages, but the taxonomic importance of these morphological characteristics was emphasized differently at the genus and species levels [5]. Morphological characteristics in spermangonia and telia have long been used for classification at the genus level, and these uredinial and telial stages were of significant importance for species delimitation [5, 11, 12]. Together with morphological characteristics in the uredinial and telial stages, host ranges have been variably emphasized for species recognition since the discovery of host alternation of rust fungus via inoculation experiments [13–16]. In the middle of the 20^th^ century, species recognition gradually came to rely upon the morphology in the uredinial and telial stages, whereas ecological attributes, such as telial or aecial host information, served as important criteria for subspecies recognition (i.e., varieties or formae speciales) [7, 12]. Since then, several previously described species that were primarily differentiated based on their host ranges in the aecial or telial stages were included into the *M*. *epitea* species complex [17]. Although species delimitation relied solely on morphology, various morphological characteristics were used for species delimitation among different taxonomic systems [7–9]. To date, these traditionally emphasized morphological characteristics have not been thoroughly evaluated, and their effectiveness for species recognition is still unknown. Thus, confusion exists concerning the number and status of taxa, and the application of names and delimitation of *Melampsora* species on willows are difficult.

In China, early reports of *Melampsora* species began in 1908. *Melampsora coleosporioides* was the first reported species on willows in northeastern China in 1913 [18, 19]. Thereafter, regional investigations were continuously performed to explore the *Melampsora* species on willows, and the number of *Melampsora* species in China recently reached 31 [20–31]. These species are primarily recognized based on several European or Japanese taxonomic systems proposed at different period; thus, species are variously circumscribed. The existence of several species reported in China was doubtful, because they were solely reported based on differences in their willow host species. Moreover, studies on the connection between the spermogonia-aecial stages and uredinial-telial stages have been rarely recorded in China. Although taxonomic discordance exists among these reported species in China due to lack of a consensus system at the national level, no taxonomic revision of *Melampsora* species on willows has been conducted. Therefore, a comprehensive taxonomic study of *Melampsora* species reported on willows in China is required.

Recently, DNA-based phylogenetic analyses have been used for the taxonomy of rust fungi, such as species from the genera *Puccinia*, *Pucciniastrum*, *Chrysomyxa*, *Phakopsora*, *Uromyces* and *Gymnosporangium* [32–36]. Molecular phylogenetic studies using the nuclear ribosomal RNA gene (rDNA) large subunit (LSU), small subunit (SSU) and internal transcribed spacer (ITS) could be informative and reveal interspecific relationships [34]. Moreover, these analyses also helped to determine the principal morphological characteristics used for species delimitation because the molecular phylogenetic results were consistent with the morphological observations [35, 37]. However, controversy in the morphological and molecular results was recognized among studies on *Melampsora* species on willows. Smith et al. [38] conducted morphological and molecular phylogenetic analyses of *M*. *epitea* from North America and recognized molecular divergence within *M*. *epitea* in the sequence data of the rDNA ITS regions. Thereafter, Bennett et al. [39] recognized 14 phylotypes within *M*. *epitea* in North America, but no clear morphological differences were identified among these phylotypes in their study. Similar results were obtained from molecular phylogenetic studies on *M*. *epitea* in Europe, *M*. *capraearum* and *M*. *epiphylla* [12, 29, 30, 40]. All of these studies revealed the discordance between morphology and molecular phylogeny. These contradictions undermine the reliability of using either the genealogical species concept or the morphological species concept for species recognition among *Melampsora* species on willows. Thus, reevaluation of species boundaries, which were delimitated based on morphological species recognition and phylogenetic species recognition, was required.

To clarify the taxonomy of *Melampsora* on willows reported in China, morphological and molecular information was verified to determine the species boundaries. For this purpose, numerical taxonomic studies were undertaken to determine the morphological species boundaries. Moreover, the generalized mixed Yule-coalescent (GMYC) model analysis was employed to delimitate phylogenetic species limits. This approach was first introduced in Pons et al. [41] and further developed by Fujisawa and Barraclough [42] to delimitate species using molecular information even with single-locus data. Based on the correlation of recognized morphological and phylogenetic species, in this study we evaluated the taxonomic effectiveness of morphological and molecular information in recognizing *Melampsora* species on willows. Additionally, we investigated the circumscription of *Melampsora* species on willows in China.

## Materials and Methods

### Fungal specimens

Two hundred and six dried specimens from China were borrowed from several herbaria to cover the largest possible host and locality range based on taxonomic literature reported in China. Most of these specimens were used for species description or illustration in Tai [43], Wang et al. [20], Zhuang [21–24], Cao and Li [44], Zhuang and Wei [26, 27], Liu [45], Zhuang [2005] and Zhao et al. [29–31]. Specimens were chosen according to the name on the attached labels and the host information. These specimens contained all 31 of the *Melampsora* species reported in China. An additional 229 specimens from Europe, Japan and Russia were borrowed for morphological and phylogenetic comparisons. These specimens were labeled either with the same name as 25 species reported in China or were from the same host species. The detailed information of all of the studied specimens will be listed in a monographic revision of *Melampsora* species on willows in China in the future.

All examined specimens were borrowed from the following herbaria: the Mycological Herbarium of Institute of Microbiology, CAS, China (HMAS); the Herbarium of the College of Forestry, Inner Mongolia Agricultural University (HNMAP); the Mycological Herbarium of College of Forestry, Northwest A & F University, China (HMNWFC); the Systematic Mycology and Microbiology Laboratory, Agricultural Research Service, USDA, USA (BPI); the Mycological Herbarium of the Graduate School of Life and Environmental Sciences, University of Tsukuba, Tsukuba, Japan (TSH); the Hiratsuka Herbarium, Tokyo, Japan (HH); and the National Museum of Nature and Science, Tsukuba, Japan (TNS). An additional 9 specimens from England were kindly supplied by Dr. Ming-Hao Pei (Rothamsted Research, Harpenden, Hertfordshire, UK).

Among these 435 dried herbarium specimens, type specimens of the following species were included: *M*. *kamikotica* (HH-73060, holotype); *M*. *larici-urbaniana* (HH-78307, neotype and HH-53302, isoneotype); *M*. *epiphylla* (HH-77578, isotype); *M*. *yezoensis* (HH-99463, neotype and HH-53165); *M*. *microsora* (HH-53150, isotype); *M*. *kiusiana* (HH-53157, holotype); *M*. *humilis* (HH-53278, isotype), *M*. *salicis-viminalis* (HMAS38658, holotype), *M*. *salicis-cavaleriei* (HMAS3607, holotype) and *M*. *tsinlingensis* (HMAS76119, holotype).

### Species delimitation based on morphological characteristics

Morphological characteristics in the uredinial and telial stages were examined under a dissecting microscope (DM) (Leica, Tokyo, Japan), light microscope (LM) (Leica, Tokyo, Japan) and scanning electron microscope (SEM) (Hitachi, Tokyo, Japan). All recognized morphological characteristics were categorized into two different types: qualitative characteristics and quantitative characteristics. Qualitative characteristics were directly checked by DM, LM and SEM, whereas the quantitative characteristics were analyzed using an image analyzer such as Q-Win Image analyzer (Leica, Tokyo, Japan) and the freely available image analysis software Photoruler ver. 1.1 (http://www.inocybe.info/_userdata/ruler/PhotoRuler.html). Fifty urediniospores, paraphyses or teliospores were randomly measured for each specimen. To define the shape of the urediniospores, a numerical data, shape factor, was analyzed by Sigma Scan Pro ver. 5.0 for Windows (SPSS Science, Chicago, IL, USA). This characteristic is a dimensionless quantity used in image analysis that numerically describes the shape of urediniospores independent size. SEM was used to examine the surface structures of sori and spores. Samples were dusted on a double adhesive tape on a specimen holder and coated with platinum-palladium at a 25 nm thickness by a Hitachi E1030 Ion Sputter (Hitachi, Tokyo, Japan). The samples were observed with an S-4200 scanning electron microscope (Hitachi, Tokyo, Japan) operated at 15 kV.

In this study, numerical taxonomic methods, cluster analysis and one-way ANOVA were used to classify the morphological species among specimens. The states of qualitative characteristics were coded into different numbers (Table 1), and the data matrix was constructed together with the mean value of quantitative characteristics (S1 Table). Higher variations of several morphological characteristics (e.g. dimensions of paraphyses, width of urediniospores and width of teliospores) were found within each specimen compared to between specimens; thus, these characteristics were excluded from further study. Cluster analysis was conducted with the software package SPSS ver. 20.0 for windows (SPSS, Chicago, IL, USA), and hierarchical clustering analysis was employed using the neighbor-joining method and Ward’s method. To reduce the effects of different scales of measurement used for different quantitative characteristics, the quantitative variables were transformed into standardized values and each value for the item being standardized was divided by the range of the values. A dendrogram was established to recognize the possible groups of *Melampsora* species on willows based on the similarity of morphological data. Finally, one-way analysis of variance (ANOVA) was used to verify morphological differences among specimens within each possible group, and morphological groups were determined until no apparent difference among the tested characteristics was recognized. Thereafter, to determine the diagnostic characteristics in each morphological group, the divisive method of cluster analysis and one-way ANOVA was conducted in each recognized cluster and subcluster in the dendrogram to progressively detect the diagnostic characters for each morphological group.

**Table 1:** Morphological character recognized in uredinial and telial stages in this study.

Spore Stage	Qualitative Characters	Quantitative Characters
**Uredinial Stage**	Position of uredinia (1: hypophyllous; 2: epiphyllous; 3: amphigenous)	Shape factor of urediniospores (0 to 1)
	Ornamentation of urediniospores (1: without smooth regions; 2: with smooth regions at apex)	Length of urediniospores
	Spine form of urediniospores (1: echinulate 1; 2: echinulate 2; 3: echinulate 3)	Wall thickness of urediniospores
	Position of germ pore (1: scattered; 2: tending to bizonate)	Mean distance between spines
	Existence of intermixed paraphyses (1: intermixed; 2: peripheral)	
	Apex of paraphyses (1: evenly thickened; 2: thickened at apex)	
**Telial Stage**	Position of telia (1: hypophyllous; 2: epiphyllous; 3: amphigenous)	Length of teliospores
	Position of teliospores (1: subepidermal; 2: subcuticular; 3: subepidermal or subcuticular)	Wall thickness of teliospores

### Species delimitation based on phylogenetic data

DNA was extracted from single uredinium from all examined specimens following the procedure of Virtudazo et al. [33]. For these older herbarium specimens, the DNA extract was diluted 50-fold or 100-fold for some old specimens to successfully amplify the target fragment. To study the phylogenetic position of each specimen, two nuclear ribosomal RNA gene regions (the D1/D2 regions of the LSU and the ITS region) were amplified according to Tian et al. [37]. After amplification, the PCR products were cut from the gel and purified with the Wizard^®^ SV Gel and PCR Clean-up Kit (Promega, Madison, WI, USA). The purified PCR products were sequenced directly using the BigDye^TM^ Terminator Cycle Sequencing Ready Reaction Kit (Applied Biosystems, Foster City, CA, USA) with the same amplification primer sets used for PCR amplification. Sequences were analyzed on a 3130 Automated DNA Sequencer (PE Applied Biosystems).

The herbarium number, host species, geographical origins and GenBank accession numbers of the sequenced specimens were indicated in the S2 Table. The raw sequence data were manually aligned with Bioedit ver. 7.0.9 [46]. Multiple alignments were performed with Clustal X ver. 1.8 [47]. Because different genes provided resolution and support in different regions of the tree, a total evidence analysis yielded the best results for the phylogeny [48]. Thus, the rDNA ITS regions and D1/D2 regions were combined together to yield the best results. Phylogenetic trees were constructed with two sequences of *M*. *laricis-populina* Kleb. as the outgroups. Maximum parsimony analysis (MP) and maximum likelihood analysis (ML) were performed using PAUP* ver. 4.0b10 [49]. Bayesian Markov chain Monte Carlo (MCMC) analysis was performed with MrBayes ver. 3.1.2 [50]. In the ML and Bayesian analyses, the best-fit substitution models were estimated by Modeltest ver. 3.7 [51].

We employed GMYC model analysis to determine the phylogenetic species boundaries. Ultrametric trees were constructed by BEAST ver. 1.7.5 [52] using the same substitution models as in the analyses performed in MrBayes. The GMYC analysis required an ultrametric phylogenetic tree constructed using unique haplotypes, thus, duplicate haplotypes and the two outgroup samples were removed using TCS ver. 1.21 [53]. We performed two sets of analyses using a single-threshold model or a multiple-threshold model, and three independent MCMC analyses were run for 100 million generations with trees sampled every 10,000 generations. The posterior tree sample was summarized using TreeAnnotator [54] after discarding the first 5000 trees of each run as the burn-in. The selected topologies were used to optimize the single- and multiple-threshold GMYC models, using the ‘splits’ package [42, 55] available for R 3.0.2 (R Core Team 2013). The STEM program was used to estimate the likelihood scores of alternative species delimitation scenarios obtained from single- and multiple-threshold GMYC [56], and the putative species scenario was selected based on the value of the estimated likelihood scores according to Carstens and Dewey [57].

## Results

### Different morphological types recognized among specimens

**Fig 1:**
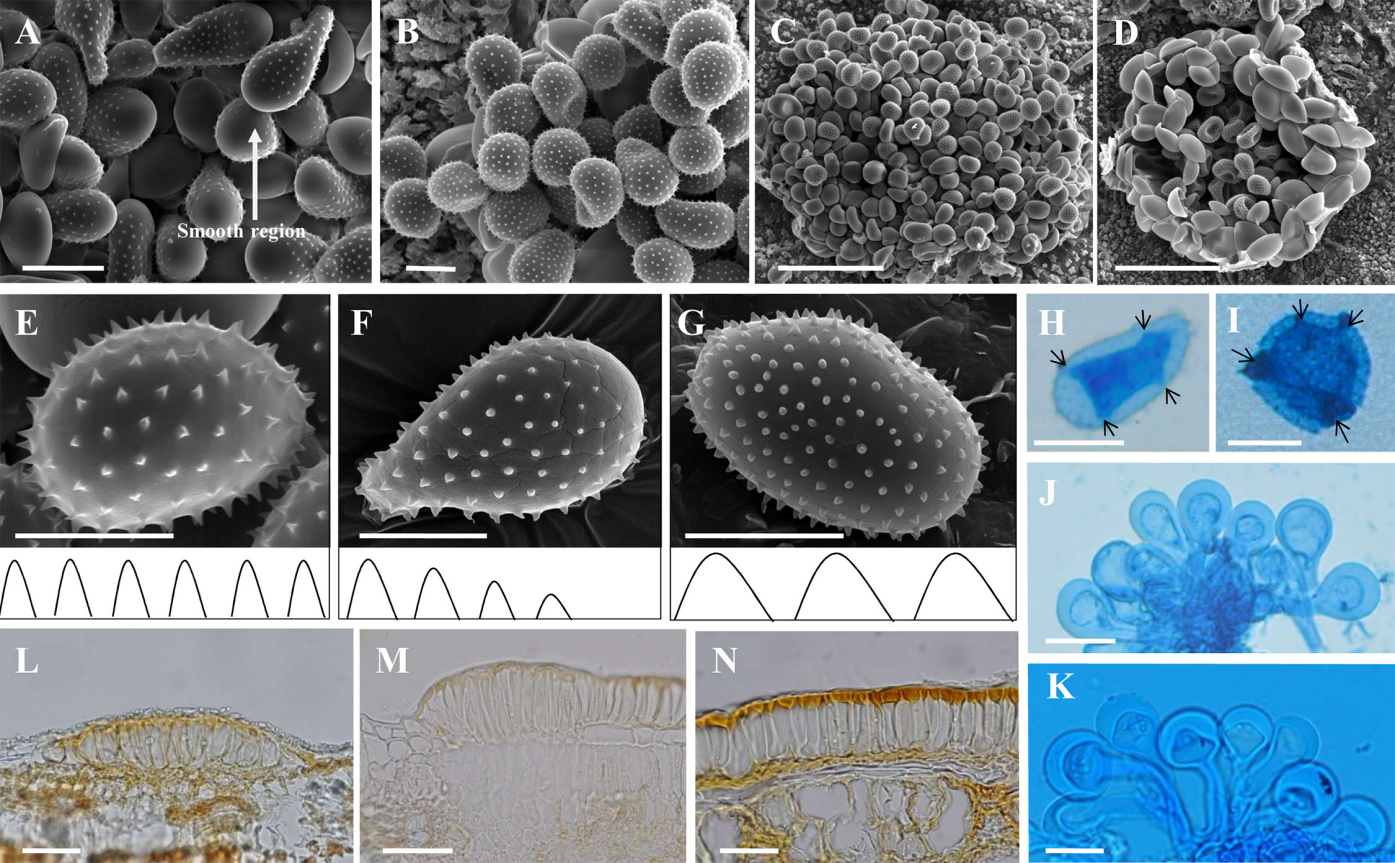
Qualitative morphological characteristics recognized in this study. (A) Urediniospores with a smooth apex. (B) Urediniospores without a smooth apex. (C) Uredinia with intermixed paraphyses. (D) Uredinia without intermixed paraphyses. (E) Urediniospores with echinulate type 1 spines. (F) Urediniospores with echinulate type 2 spines. (G) Urediniospores with echinulate type 3 spines. (H) Urediniospores with biozonate germ pores. (I) Urediniospores with scattered germ pores. (J) Paraphyses with evenly thickened membranes. (K) Paraphyses with an apparently thickened apex. (L) Subepidermal teliospores. (M) Subepidermal or subcuticular teliospores. (N) Subcuticular teliospores. Bars: A, H, J, K, M, N = 20 mm; B, F, G, I = 10 mm; C = 50 μm; D = 60 μm; E = 5 μm; L = 30 μm.

Qualitative characteristics in the uredinial and telial stages were categorized into different types based on morphological observations of 435 specimens using DM, LM and SEM. In the uredinial stage, the position of the uredinia was categorized into three types: amphigenous, epiphyllous and hypophyllous (Fig 1A and 1B). Specimens were divided into two different types based on the existence of a smooth region in the urediniospores: specimens with a smooth region or smooth spot at the apex and specimens without a smooth region or spot at the apex (Fig 1C and 1D). The position of the paraphyses of the specimens was divided into two types based on ultrastrucutural observations of the uredinia: uredinia with intermixed paraphyses and uredinia with peripheral paraphyses (Fig 1E and 1F). Based on ultrastructural observations by SEM, the morphology of spines on the urediniospores of the examined specimens could be separated into three different forms: echinulate type 1, echinulate type 2 and echinulate type 3 (Fig 1G–1I). Echinulate type 1 was characterized by the even distribution of stout, sharp-pointed conical spines; the spine form of most of the specimens fell into this category. Echinulate type 2 was characterized by gradually decreased spines towards the smooth area on the urediniospores. Echinulate type 3 was characterized by conical, straight or slightly curved spines on the surface of the urediniospores. Two distinctive types of germ pores were recognized among all of the examined specimens. Some specimens had scattered germ pores, whereas other specimens possessed germ pores tending to biozonate (Fig 1J and 1K). The apex of the paraphyses of the examined specimens could be separated into two types. One type had paraphyses with evenly thickened membranes at the side and apex, whereas the second type had paraphyses that were apparently thickened at the apex (Fig 1L and 1M). In the telial stage, three types of positions of the telia, amphigenous, epiphyllous and hypophyllous were observed. The positions of the teliospores of all examined specimens were classified into three distinctive types: subepidermal, subcuticular or both subepidermal and subcuticular teliospores together (Fig 1N–1P).

### Morphological species recognition

**Fig 2:**
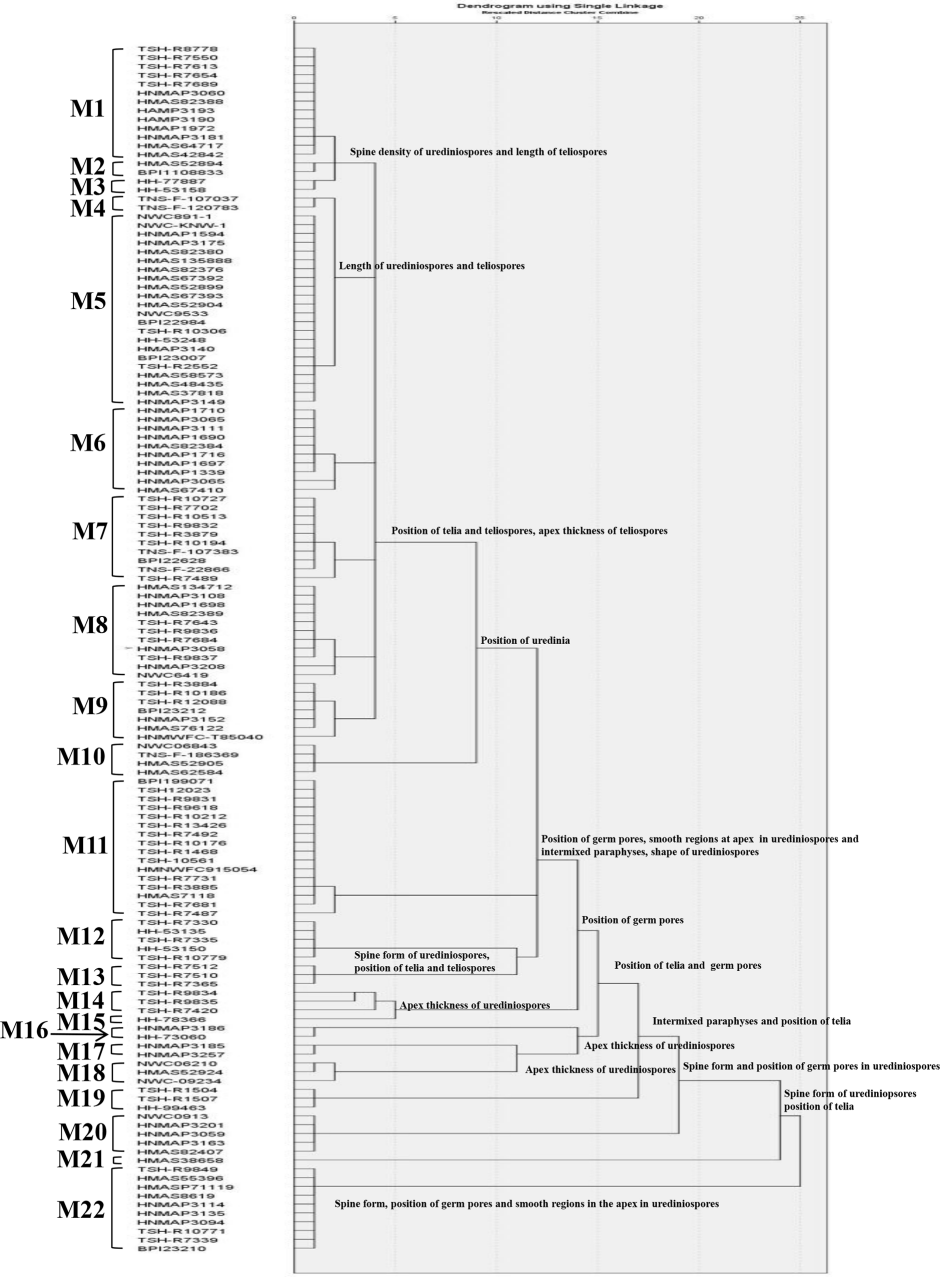
Dendrogram resulting from 14 morphological characteristics of 137 *Melampsora* specimens on willows. The specimens were divided into 22 groups (M1 to M22) based on the similarities of these characteristics. Based on a hierarchical clustering analysis using a divisive method, each branch was divided based on the morphological characteristics indicated on the node.

To compare the morphological and molecular results, 137 specimens that possessed both uredinial and telial morphology and sequence data, were used for numerical taxonomic analyses. The detailed information of these specimens is shown in the S2 Table. A total of 22 groups were recognized by hierarchical clustering analysis (Fig 2); the results from one-way ANOVA indicated that no apparent differences were recognized among the specimens within each group. Thus, these groups were recognized as separate morphological species designated M1 to M22. Among these 22 morphological groups, 16 morphological groups conformed to 16 existing species [*M*. *salicis-argyraceae* (M2), *M*. *kiusiana* (M3), *M*. *salicis-sinicae* (M6), *M*. *capraearum* (M7), *M*. *ribesii-viminalis* (M8), *M*. *microsora* (M12), *M*. *chelidonii-pierotii* (M13), *M*. *larici-urbaniana* (M14), *M*. *chosenia* (M15), *M*. *kamikotica* (M16), *M*. *tsinlingensis* (M17), *M*. *salicis-albae* (M18), *M*. *yezoensis* (M19), *M*. *laricis-pentandrae* (M20), *M*. *salicis-viminalis* (M21) and *M*. *coleosporides* (M22)] that fitted well with the original descriptions and type specimen morphologies [7, 9, 12, 16, 30, 31, 58, 59]. However, specimens identified as *M*. *epitea* [9] were located in 5 morphological groups (M1, M4, M5, M10 and M11). Similarly, specimens recognized as *M*. *epiphylla* [9] were located in two morphological groups (M2 and M9).

We applied the divisive method of cluster analysis together with one-way ANOVA to determine the diagnostic characteristics used to differentiate these morphological species in the dendrogram. The diagnostic characteristics based on morphological comparison of each cluster or group from the initial step when separation of cluster began were shown on the node of the dendrogram in the Fig 2. These 14 recognized characteristics were sufficient to separate these morphological species at different similarity levels. These characteristics comprised 8 qualitative characteristics (the existence of smooth regions in urediniospores, the spine form of urediniospores, the existence of intermixed paraphyses, the position of the germ pore, the existence of a thickened apex in the paraphyses the position of the uredinia, the position of the telia and the position of the teliospores) and 6 quantitative characteristics (the shape factor of urediniospores, the wall thickness of urediniospores, the spine density of urediniospores, the length of urediniospores, the length of teliospores and the apex thickness of teliospores).

### Molecular phylogenetic species recognition

**Fig 3:**
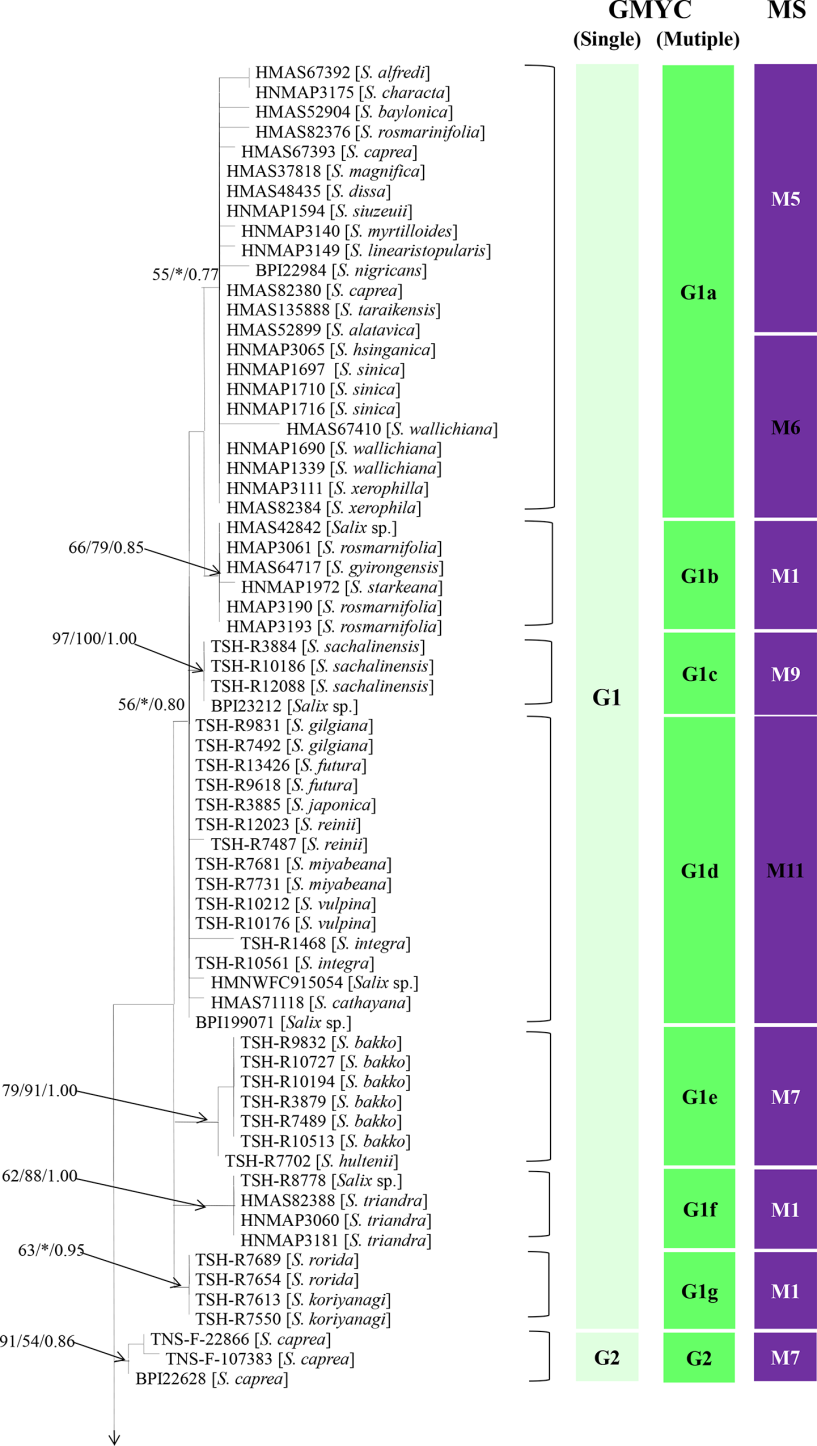
Phylogenetic trees of the combined data of the rDNA ITS regions and D1/D2 regions obtained from parsimony analysis. Bayesian posterior probabilities (Bpp) were given immediately followed by the bootstrap values from MP and ML on the nodes in the topology. Asterisks (*) represent bootstrap values less than 50% or Bpp less than 0.75 in the topology. The first column depicts species recognized by the single-threshold GMYC model, and the second column depicts putative species recognized by multiple-threshold model. The third column depicts morphological species recognized by numerical taxonomy.

**Fig 4:**
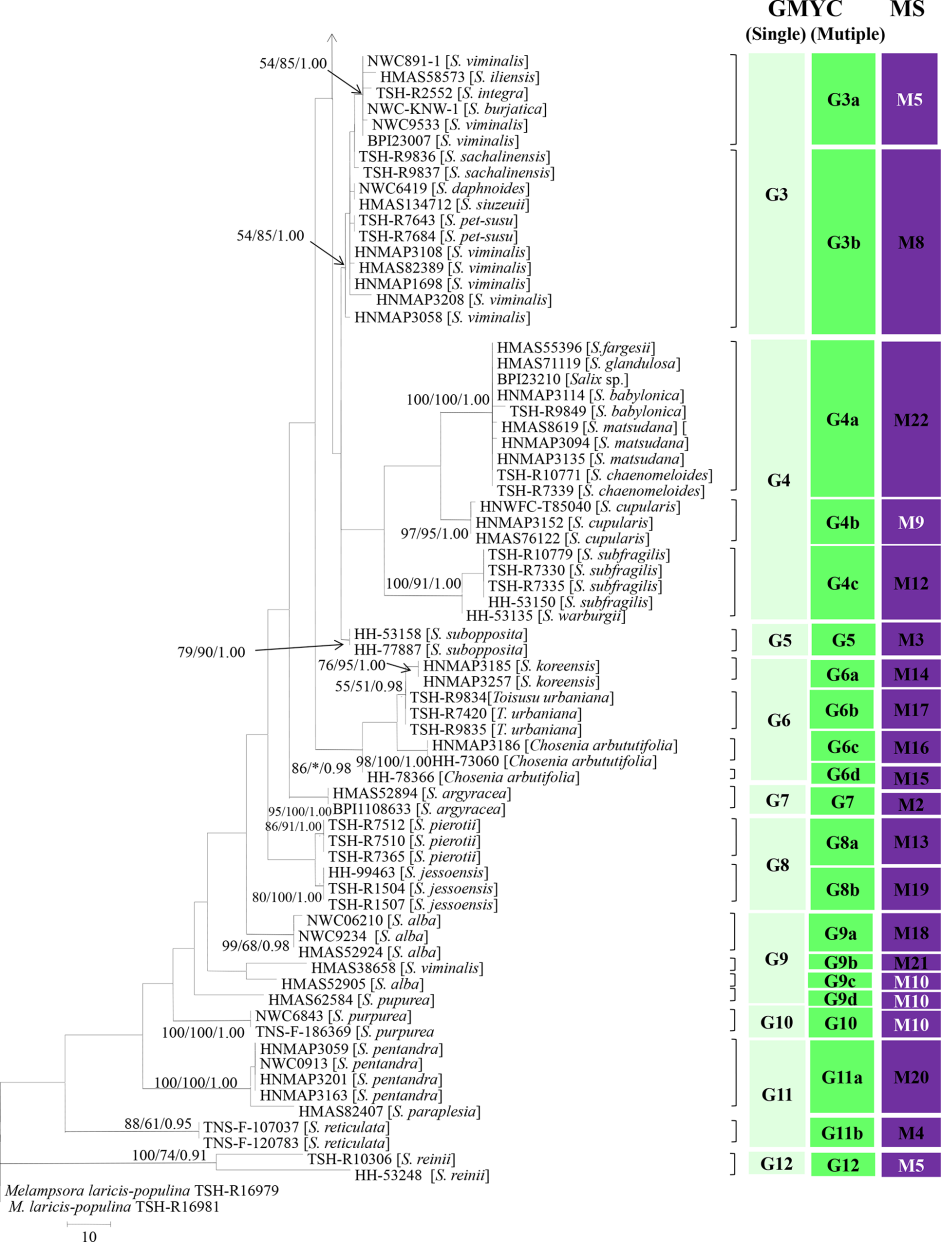
Continuous part of the phylogenetic trees of the combined data from the rDNA ITS regions and D1/D2 regions obtained from parsimony analysis.

Sequences were successfully amplified from the rDNA ITS regions and D1/D2 regions from 137 specimens. The whole sequence data matrix of the rDNA ITS regions and D1/D2 regions ranged from 1051 bp to1128 bp, and 156 sites among 232 variable sites were parsimony informative. The best-fit evolutionary model selected by Modeltest was TVM+I+G. The phylogenetic tree constructed by MP, ML and Bayesian inference was illustrated in Figs 3 and 4.

We applied the GMYC approach to identify the phylogenetic species using the rDNA ITS region and D1/D2 region sequences. A total of 73 haplotypes were found among 137 specimens, and both the single-threshold and multiple-threshold models resulted in a significantly better fit to the ultrametric tree compared to the null model. However, the GMYC analyses revealed different results for the single- and multiple- threshold models. Based on the single-threshold model, all of these specimens were categorized into 12 putative species designated G1 to G12 (Figs 3 and 4). However, the multiple-threshold model supported a 29-species scenario. Among these recognized putative species, only four putative species (G2, G5, G7 and G10) were recognized by both approaches. However, other putative species from the single-threshold model were further split into several putative species. The multiple-threshold model was preferred over the single threshold model because most of the putative species derived by the multiple-threshold mode were supported by the Bayesian, MP and ML analyses.

Thereafter, two possible species delimitation scenarios were evaluated using STEM ver. 2.0 [56] based on the protocol of Carstens and Dewey [57]. We compared a 12-species scenario, a 29-species scenario, and a 1-species scenario. An information-theoretic approach that accommodated numbers of parameters strongly supported the 29-species scenario (S3 Table). Thus, we followed the results from the GMYC and STEM evaluation and recognized the grouping of 137 specimens into 29 putative phylogenetic species.

### Correlation of the morphological and molecular phylogenetic species recognition

Numerical taxonomy of the morphological characteristics revealed 22 morphological species (M1 to M22) that corresponded to 29 putative phylogenetic species based on sequence data of the rDNA ITS regions and D1/D2 regions. Among them, 16 morphological species (M2, M3, M4, M8, M11, M12, M13, M14, M15, M16, M17, M18, M19, M20, M21 and M22) were consistent with the phylogenetic species. However, 6 other morphological species were split into two or more phylogenetic species. Among them, M5, M7 and M9 corresponded to two phylogenetic species. M1 and M10 corresponded to three phylogenetic species. M6 and M5 corresponded to only one phylogenetic species (G1). The correlations between the morphological species and phylogenetic species were shown in S1 Table.

Based on the morphological comparison of specimens from each morphological species in the different phylogenetic groups, we recognized subtle morphological differences in the uredinial and telial stages (S1 Table). For example, specimens of M7 from two phylogenetic species (G1e and G2) showed subtle morphological differences in the distance between spines and the length of teliospores. Subtle differences were also found in two phylogenetic species (G1c and G4b) that were recognized as M9. Similar results were also recognized in M10, although these differences were not recognized by numerical taxonomy. Thus, subtle morphological differences in the distance between spines and length of teliospores could differentiate between these cryptic species. Moreover, although M5 was further split into three phylogenetic species, no clear morphological differences were recognized with the exception of the telial host ranges. Among these three phylogenetic species, G1a (M5) was found on *S*. *alfredii*, *S*. *caprae*, *S*. *character*, *S*. *dissa*, *S*. *purpurea*, *S*. *linearistopularis*, *S*. *myrtilloides*, *S*.*magnifica*, *S*. *taraikensis*, *S*. *rorida* and *S*. *rosmarinifolia*, G3a (M5) was found on *S*. *iliensis* from China, *S*. *burjatica* and *S*. *viminalis* from Europe and *S*. *integra* from Japan. G12 (M5) was found on *S*. *reinii*.

## Discussion

### Species delimitation based on integrative information of morphological characteristics and molecular data

Since the establishment of the genus *Melampsora*, several different species concepts have been employed to define species on willows [5, 9, 17]. Among them, the morphological species concept was the dominant operational species concept, and therefore species were mainly described and diagnosed based on morphological characteristics [5]. Recently, phylogenetic species recognition has been increasingly used for species delimitation in the genus *Melampsora*, especially cryptic species that are resistant to traditional morphological species concepts [38, 39]. In this study, we report the high concordance of species recognition based on morphology and molecular data. However, we also found discordance in morphological and molecular species boundaries. The correlation of species recognized by the two approaches indicated that phylogenetic species recognition seemed to be more effective in recognizing cryptic lineages, such as morphological species M5. Although no clear morphological differences in the uredinial and telial stages were recognized, M5 was further split into three phylogenetic species (G1a, G3a and G12). Based on host information from two phylogenetic species, previous taxonomic descriptions and inoculation experiments [7–9], the aecial host species and aecial morphology could be presumed to play an important role in delimitating these three species. In contrast, sometimes morphological species recognition seemed to be sensitive to certain taxa (i.e. M5 and M6, which were recognized as same phylogenetic species based on rDNA sequence data). This discordance was caused by insufficient molecular information, because the separation of M5 and M6 was demonstrated using the translation elongation factor 1α gene in our previous study [30]. Therefore, the above-mentioned discordance of morphological and molecular data was caused by limited sampling of certain taxa or limited sequence data obtained from specific DNA loci; the divergence of either morphological or molecular data seemed to be effective and operational for species recognition. Our results indicated that integrating both morphological and molecular data was a good approach for identifying and delimiting independent lineages. This integrative taxonomy, which was previously proposed by Will et al. [60], proved to be applicable for estimation of the diversity of *Melampsora* species on willows in China.

At present, two different integrative taxonomy methods exist: ‘integration by congruence’ and ‘integration by cumulation’ [61]. The approach of ‘integration by congruence’ was adopted in accordance with the assumption that concordant patterns of divergence among several taxonomic characteristics indicated full lineage separation. This method was widely used for taxonomic studies in rust fungi, such as taxonomic studies of *Melampsora* species on poplar, *Pucciniastrum* species, and *Phakopsora* species [35, 36, 37]. However, it has the risk of underestimating species numbers because the process of speciation is not always accompanied by characteristic changes at all levels [61]. Another approach in integrative taxonomy is ‘integration by cumulation’, which is based on the assumption that divergences in any taxonomic characteristics can provide evidence for the existence of a species [62]. The recognition of a species is decided based on the available information which is considered to be a good indicator of lineage divergence; thus, this method is probably most suitable to uncover recently diverged species in adaptive radiations [63, 64]. In this study, the correlation of morphological and molecular data suggested that the ‘integration by cumulation’ method should be applied to determine the *Melampsora* taxa on willows. This approach was used for taxonomic studies on rust fungi for the first time and provided the best resolution for distinguishing species based on both concordance and discordance of the morphological and molecular data.

### Effectiveness of numerical taxonomy for morphological species delimitation and diagnostic characteristic selection

Few researchers have used numerical methods in fungal taxonomy due to the fear of the mathematical problems involved in the presence of mixed-type (continuous and categorical) data originating from their investigations [65]. However, numerical taxonomy has the advantage of implementing quantitative assessment of trait variation for species delimitation, and it enables the selection of diagnostic characteristics capable of differentiating between different clusters based on the frequency of positive characteristics occurring in each group with the aid of the computer program [66, 67]. In this study, the numerical taxonomic method was implemented to detect possible morphological groups in *Melampsora* species on willows. Additionally, the numerical taxonomic method can also be used to calculate the frequency of the positive characteristics occurring in each recognized group. Cluster analysis is effective at determining one or several diagnostic characteristics that should be selected as the main characteristics for species identification and taxonomy [66, 67]. However, the numerical taxonomic method had some limitations in distinguishing morphologically cryptic taxa with minute morphological differences, especially those with a limited number of specimens. Our morphological and molecular studies revealed that these limitations in species recognition could be overcome with the aid of molecular data.

### Evaluation of morphological criteria for species recognition

Based on the morphological comparison and dendrogram obtained from our cluster analysis, 14 morphological characteristics were shown to possess diagnostic characteristics capable of differentiating 22 morphological groups, because most of the species recognized by these characteristics were supported by the molecular data. With the exception of the distance between spines, the spine form of urediniospores, the shape factor of urediniospores and the existence of intermixed paraphyses, the other diagnostic characteristics have served as important criteria for species recognition for a long time [12–15, 44, 45, 58, 59]. Among these four newly recognized characteristics, the distance between spines and the spine form of urediniospores were previously used for species recognition of other related rust fungi, such as *Melampsora* species on poplar, *Gymnosporangium*, *Pucciniastrum*, *Phragmidium*, *Puccinia*, and *Phakopsora* [33, 36, 37, 68, 69]. However, the factor of urediniospores and the existence of intermixed paraphyses were demonstrated to be effective taxonomic criteria in rust fungi for the first time. The shape of urediniospores was frequently used for species recognition, and several different types (i.e., globoid, ellipsoid, ovoid and obovoid to broadly ellipsoid) were described in several taxonomic systems [9, 12, 59]. However, it was difficult to recognize these different types, because the terminology and definitions used to describe the shape were not precise. Here, we employed the shape factor as a numerical quantity to precisely determine the shape of urediniospores, and this parameter was demonstrated to be an effective characteristic. Although this characteristic was suggested to be an important characteristic based on the results of the molecular phylogenetic studies of *M*. *epitea* in North America [38], it was demonstrated to be an important taxonomic criterion for species recognition for the first time in this study. The uredinia of *Melampsora* species were recorded as the *Uredo*-type with intermixed paraphyses based on the classification of Cummins and Hiratsuka [11]. However, two different types of uredinia were found based on the position of paraphyses through careful morphological observation of large amounts of specimens by SEM. One type of uredinia possessed intermixed and peripheral paraphyses, whereas the other type possessed uredinia with peripheral paraphyses (Fig 1C and 1D). The former type is frequently reported in the genus *Melampsora*. The other type is similar to the *Calidion*-type, and this type has not previously been reported in genus *Melampsora*. The position of the paraphyses in uredinia was demonstrated to be a new and stable characteristic for the recognition of *Melampsora* species on willows.

### Host specificity

In the early 20th century, host range was employed for species recognition when the life cycle of *Melampsora* species on willows was discovered [13–15]. Based on extensive inoculation experiments, Klebahn redefined the taxonomy of *Melampsora* species on willows based on the morphology and host ranges of both the aecial and telial stages. This taxonomic treatment was generally accepted by Schneider [70], Matsumoto [71], Sydow and Sydow [16], Arthur [72] and other taxonomists at the beginning of the 20^th^ century. This taxonomic treatment was previously accepted by Chinese taxonomists, although the life cycle information of these reported *Melampsora* species was not verified in China. In this study, we chose specimens on different willow sections; however, no correlation between willow sections and *Melampsora* species was found, because some morphologically and phylogenetically distinct species shared the same host species. Specimens on several willow sections were placed in the same phylogenetic species (i.e., phylogenetic species G1a, which included rust collections on willow sections of *Haoanae*, *Magnificae*, *Vetrix*, *Daphnella*, *Helix*, *Mytilloides*, *Wilsonianae*, *Vimen*, *Salix* and *Tetraspermae*). Moreover, some rust specimens on the same willow sections were scattered into several distinct species. For example, rust specimens on host *S*. *viminalis* were recognized as M5, M8 and M21. Similar situations were found in rust specimens on *S*. *triandra*, *S*. *alba*, *S*. *caprea* and *S*. *purpurea*, and host specificity did not reflect any correlation with the *Melampsora* species among these species. The ecological species concept using telial host ranges for species recognition was not suitable for *Melampsora* species on willows, especially in China, which had large amounts of willow species (over 264 species) [73].

### Recognition of taxa in this study

The names of 17 taxa were confirmed based on the type specimens, morphology and molecular analyses. They were confirmed as *M*. *salicis-argyraceae* (M2), *M*. *kiusiana* (M3), *M*. *salicis-sinicae* (M6), *M*. *ribesii-viminalis* (M8), *M*. *microsora* (M12), *M*. *chelidonii-pierotii* (M13), *M*. *larici-urbaniana* (M14), *M*. *chosenia* (M15), *M*. *kamikotica* (M16), *M*. *tsinlingensis* (M17), *M*. *salicis-albae* (M18), *M*. *yezoensis* (M19), *M*. *laricis-pentandrae* (M20), *M*. *salicis-viminalis* (M21) and *M*. *coleosporides* (M22). However, the other recognized taxa could not be designated to certain *Melampsora* species due to the lack of type specimens of *M*. *epitea*, *M*. *capraearum* and their synonyms. Thus, further studies need to be conducted to confirm the taxonomic identities of these *Melampsora* species, and a monographic revision of *Melampsora* species on willows in China will be published in future.

## Supporting Information

S1 Table Morphological variations of specimens in the uredinial and telial stages from different species. (XLS)

S2 Table Host plant, locality and herbarium number of rust specimens in different taxa used for molecular phylogenetic analyses. (DOCX)

S3 Table Species delimitation scenarios in the molecular data and likelihood scores for the STEM analysis of species delimitation scenarios (k = number of parameters, high ⊿lnL indicates high support for a given scenario). (DOCX)
