# Supplementary material for: Integrating a Numerical Taxonomic Method and Molecular Phylogeny for Species Delimitation of Melampsora Species (Melampsoraceae, Pucciniales) on Willows in China
Source: PLoS One. 2015 Dec 17;10(12):e0144883. doi: 10.1371/journal.pone.0144883 (PMC4683050; doi:10.1371/journal.pone.0144883)
Supplement: S2 Table — (DOCX) [file pone.0144883.s002.docx]

| **Host plants** | **Voucher specimens^a^** | **Locality** | **ITS** | **D1/D2** |
| --- | --- | --- | --- | --- |
| *Chosenia arbutifolia* | HNMAP3186 | China, Inner Mongolia | KF780760 | KF780643 |
| *C. arbutifolia* | HH-73050 | Japan, Nagano | KF780842 | KF780623 |
| *C. arbutifolia* | HH-78366 | Russia | KF780836 | KF780719 |
| *S. alba* | HMAS52905 | China, Xinjiang | KF780741 | KF780624 |
| *S. alba* | NWC-09234 | England, Rothamsted | KF780774 | KF780657 |
| *S. alba* | NWC-06210 | England, Rothamsted | KF780757 | KF780640 |
| *S. alba* | HMAS52924 | China, Xinjiang | KF780803 | KF780686 |
| *S. alfredi* | HMAS67392 | China, Hubei | KF780771 | KF780654 |
| *S. argyracea* | HMAS52984 | China, Xinjiang | KF780733 | KF780616 |
| *S. argyracea* | BPI1108633 | China, Tibet | KF780803 | KF780686 |
| *S. babylonica* | HMAP3114 | China, Inner Mongolia | KF780755 | KF780638 |
| *S. babylonica* | TSH-R9849 | Japan, Nagano | KF780776 | KF780659 |
| *S. bakko* | TSH-R9832 | Japan, Nagano | KC631851 | KC685608 |
| *S. bakko* | TSH-R10727 | Japan, Nikko | KC631852 | KC685609 |
| *S. bakko* | TSH-R10194 | Japan, Aomori | KC631853 | KC685610 |
| *S. bakko* | TSH-R3879 | Japan, Shizuoka | KC631854 | KC685611 |
| *S. bakko* | TSH-R10513 | Japan, Gunma | KC631855 | KC685612 |
| *S. bakko* | TSH-R7489 | Japan, Nagano | KC631850 | KC685607 |
| *S. burjatica* | NWC-KNW-1 | England, Rothamsted | KF164455 | KF164449 |
| *S. caprea* | HMAS67393 | China, Hubei | KF780736 | KF780619 |
| *S. caprea* | HMAS82380 | China, Inner Mngolia | KF780770 | KF780653 |
| *S. caprea* | TNS-F-107383 | Finland | KF780842 | KF780725 |
| *S. caprea* | BPI22628 | Australia | KF780843 | KF780726 |
| *S. caprea* | TNS-F-222866 | Germany | KF780841 | KF780724 |
| *S. cathayana* | HMAS71118 | China, Sichuan | KF780819 | KF780702 |
| *S. chaenomeloides* | TSH-R10771 | Japan, Ibaraki | KF780826 | KF780709 |
| *S. chaenomeloides* | TSH-R7339 | Japan, Ibaraki | KF780835 | KF780718 |
| *S. characta* | HNMAP3175 | China, Inner Mngolia | KF780775 | KF780658 |
| *S. cupularis* | HMAS76122 | China, Shaanxi | KF780752 | KF780635 |
| *S. cupularis* | HNMWFC-T85040 | China, Shaanxi | KF780754 | KF780637 |
| *S. cupularis* | HNMAP3152 | China, Inner Mongolia | KF780824 | KF780707 |
| *S. dissa* | HMAS48435 | China, Sichuan | KF780743 | KF780626 |
| *S. fargesii* | HMAS55396 | China, Hubei | KF780764 | KF780647 |
| *S. futura* | TSH-R9620 | Japan, Niigata | KC631860 | KC685617 |
| *S. futura* | TSH-R9618 | Japan, Niigata | KF780794 | KF780677 |
| *S. futura* | TSH-R13426 | Japan, Akita | KF780793 | KF780676 |
| *S. futura* | TSH-R12057 | Japan, Yamanashi | KF780779 | KF780662 |
| *S. gilgiana* | TSH-R9831 | Japan, Nagano | KC631841 | KC685598 |
| *S. gilgiana* | TSH-R7492 | Japan, Nagano | KF780768 | KF780651 |
| *S. glandulosa* | HMAS71119 | China, Shaanxi | KF780769 | KF780652 |
| *S. gyirongensis* | HMAS64717 | China, Tibet | KF780763 | KF780646 |
| *S. hsinganica* | HNMAP3065 | China, Inner Mongolia | KF780817 | KF780700 |
| *S. hultenii* | TSH-R7702 | Japan, Hokkaido | KF780825 | KF780708 |
| *S. iliensis* | HMAS58573 | China, Xinjiang | KF780758 | KF780641 |
| *S. integra* | TSH-R2552 | Japan, Nagano | KF780759 | KF780642 |
| *S. intergra* | TSH-10561 | Japan, Tochigi | KF780860 | KF780843 |
| *S. intergra* | TSH-R1468 | Japan, Toyoma | KF780791 | KF780674 |
| *S. japonica* | TSH-R3885 | Japan, Shizuoka | KF780780 | KF780663 |
| *S. jessoensis* | TSH-1504 | Japan, Nagano | KF780806 | KF780731 |
| *S. jessoensis* | TSH-R1507 | Japan, Nagano | KF780832 | KF780715 |
| *S. jessoensis* | HH-99463 | Japan, Hokkaido | KF780833 | KF780730 |
| *S. koreensis* | HNMAP3185 | China, Inner Mongolia | KF780748 | KF780631 |
| *S. koreensis* | HNMAP3257 | China, Inner Mongolia | KF780749 | KF780632 |
| *S. koriyanagi* | TSH-R7613 | Japan, Miyagi | KF780811 | KF780694 |
| *S. koriyanagi* | TSH-R7550 | Japan, Miyagi | KF780812 | KF780695 |
| *S. linearistipularis* | HNMAP3149 | China, Inner Mngolia | KF164459 | KF164452 |
| *S. magnifica* | HMAS37818 | China, Xinjiang | KF780742 | KF780625 |
| *S. matsudana* | HMAS8619 | China, Hubei | KF780739 | KF780622 |
| *S. matsudana* | HNMAP3094 | China, Inner Mngolia | KF780814 | KF780697 |
| *S. matsudana* | HNMAP3135 | China, Inner Mngolia | KF780823 | KF780706 |
| *S. miyabana* | TSH-R7731 | Japan, Hokkaido | KF780828 | KF780711 |
| *S. miyabana* | TSH-R7681 | Japan, Hokkaido | KF780804 | KF780687 |
| *S. myrtilloides* | HNMAP3140 | China, Inner Mngolia | KF164458 | KF164451 |
| *S. nigricans* | BPI22984 | Russia | KF780838 | KF780721 |
| *S. paraplesia* | HMAS42407 | China, Inner Mongolia | KF780762 | KF780645 |
| *S. pentandra* | NWC-0913 | England, Rothamsted | KF780756 | KF780639 |
| *S. pentandra* | HNMAP3201 | China, Inner Mongolia | KF780801 | KF780684 |
| *S. pentandra* | HNMAP3163 | China, Inner Mongolia | KF780751 | KF780634 |
| *S. pentandra* | HNMAP3059 | China, Inner Mongolia | KF780745 | KF780628 |
| *S. pet-susu* | TSH-R7643 | Japan, Hokkaido | KC631861 | KC685618 |
| *S. pet-susu* | TSH-R7684 | Japan, Hokkaido | KC631863 | KC685620 |
| *S. pierotii* | TSH-R7510 | Japan, Miyagi | KF780798 | KF780681 |
| *S. pierotii* | TSH-R7512 | Japan, Miyagi | KF780797 | KF780680 |
| *S. pierotii* | TSH-R7365 | Japan, Kagoshima | KF780799 | KF780682 |
| *S. purpurea* | HMAS62584 | China, Shandong | KF780766 | KF780649 |
| *S. purpurea* | NWC-06843 | England, Rothamsted | KF780830 | KF780713 |
| *S. purpurea* | TNS-F-186369 | Germany | KF780831 | KF780714 |
| *S. reinii* | TSH-R10306 | Japan, Tochigi | KF780777 | KF780660 |
| *S. reinii* | HH-53248 | Japan | KF780840 | KF780723 |
| *S. reinii* | TSH-R12023 | Japan, Yamanashi | KF780781 | KF780664 |
| *S. reticulata* | TNS-F-107037 | Finland, Kuusamp | KF780844 | KF780727 |
| *S. reticulata* | TNS-F-120783 | Finland, Kuusamp | KF780846 | KF780729 |
| *S. rorida* | TSH-R7689 | Japan, Hokkaido | KF780806 | KF780689 |
| *S. rorida* | TSH-R7654 | Japan, Hokkaido | KF780815 | KF780698 |
| *S. rosmarinifolia* | HMAS82376 | China, Inner Mngolia | KF780735 | KF780618 |
| *S. rosmarnifolia* | HNAMP3190 | China, Inner Mongolia | KF780767 | KF780650 |
| *S. rosmarnifolia* | HNAMP3193 | China, Inner Mongolia | KF780768 | KF780651 |
| *S. sachalinensis* | TSH-R9836 | Japan, Tochigi | KC631865 | KC685622 |
| *S. sachalinensis* | TSH-R9837 | Japan, Nagano | KC631866 | KC685623 |
| *S. sachalinensis* | TSH-R3884 | Japan, Shizuoka | KF780787 | KF780670 |
| *S. sachalinensis* | TSH-R10186 | Japan, Shizuoka | KF780788 | KF780671 |
| *S. sachalinensis* | TSH-R12280 | Japan, Yamagata | KF780789 | KF780672 |
| *S. sinica* | HNMAP1716 | China, Inner Mongolia | KC63184 | KC685601 |
| *S. sinica* | HNMAP1697 | China, Inner Mongolia | KC631845 | KC685602 |
| *S. sinica* | HNMAP1710 | China, Inner Mongolia | KC631839 | KC685596 |
| *S. siuzeuii* | HMAS134712 | China, Hei Long Jiang | KF780740 | KF780623 |
| *S. siuzevii* | HNMAP1594 | China, Inner Mngolia | KF780818 | KF780701 |
| *S. starkeana* | HNMAP1972 | China, Inner Mongolia | KF780765 | KF780648 |
| *S. starkeana* | HNMAP3176 | China, Inner Mongolia | KC631846 | KC685603 |
| *S. subfragilis* | TSH-R10079 | Japan, Ibaraki | KF780827 | KF780710 |
| *S. subfragilis* | TSH-R7330 | Japan, Ibaraki | KF780821 | KF780811 |
| *S. subfragilis* | TSH-R7335 | Japan, Hiroshima | KF780833 | KF780716 |
| *S. subfragilis* | HH-53150 | Japan, Tokyo | KF780834 | KF780717 |
| *S. subopposita* | HH-77887 | Japan, Fukuoka | KF780808 | KF780691 |
| *S. subopposita* | HH-53157 | Japan, Fukuoka | KF780807 | KF780690 |
| *S. taraikensis* | HMAS135888 | China, Hei Long Jiang | KF780813 | KF780696 |
| *S. triandra* | HNMAP3060 | China, Inner Mongolia | KF780750 | KF780633 |
| *S. triandra* | HNMAP3181 | China, Inner Mongolia | KF780829 | KF780712 |
| *S. triandra* | HMAS82388 | China, Inner Mongolia | KF780821 | KF780704 |
| *S. viminalis* | HMAS38658 | China, Tibet | KF780732 | KF780615 |
| *S. viminalis* | NWC891-1 | England, Rothamsted | KF164457 | KF164450 |
| *S. viminalis* | NWC-9533 | England, Rothamsted | KF164456 | KF164448 |
| *S. viminalis* | BPI23007 | England, Rothamsted | KF780839 | KF780722 |
| *S. viminalis* | HNMAP3108 | China, Inner Mongolia | KF780746 | KF780629 |
| *S. viminalis* | HNMAS82389 | China, Inner Mongolia | KF780773 | KF780656 |
| *S. viminalis* | HNMAP3058 | China, Inner Mongolia | KF780800 | KF780683 |
| *S. viminalis* | HNMAP3218 | China, Inner Mongolia | KF780796 | KF780679 |
| *S. viminalis* | HNMAP1698 | China, Inner Mongolia | KF780795 | KF780678 |
| *S. vulpina* | TSH-R10176 | Japan, Aomori | KF780786 | KF780669 |
| *S. vulpina* | TSH-R10212 | Japan, Aomori | KF780785 | KF780668 |
| *S. wallichiana* | HNMAP1690 | China, Inner Mongolia | KC631849 | KC685606 |
| *S. wallichiana* | HNMAP1339 | China, Inner Mongolia | KC631857 | KC685614 |
| *S. warburgii* | HH-53135 | China, Taiwan | KF780837 | KF780720 |
| *S. xerophila* | HMAS82384 | China, Heilongjiang | KC631847 | KC685604 |
| *S. xerophila* | HNMAP3111 | China, Inner Mongolia | KC631843 | KC685600 |
| *S.reinii* | TSH-R7487 | Japan, Nagano | KF780755 | KF780732 |
| *Salix* sp. | HMAS42842 | China, Inner Mongolia | KF780737 | KF780620 |
| *Salix* sp. | HNMAP3061 | China, Inner Mongolia | KF780744 | KF780627 |
| *Salix* sp. | TSH-R8778 | Russia | KF780820 | KF780703 |
| *Salix* sp. | HMAS52904 | China, Xinjiang | KF780734 | KF780617 |
| *Salix* sp. | BPI023212 | China, Anhui | KF780790 | KF780673 |
| *Salix* sp. | BPI199071 | China, Qinghai | KF780792 | KF780675 |
| *Salix* sp. | HMNWFC915054 | China, Shaanxi | KF780784 | KF780667 |
| *Toisusu urbaniana* | TSH-R9834 | Japan, Nagano | KF164453 | KF164446 |
| *T. urbaniana* | TSH-R9835 | Japan, Nagano | KF164454 | KF164447 |
| *T. urbaniana* | TSH-R7420 | Japan, Hokkaido | KF780778 | KF780661 |

* (—) indicated sequence data was failed to obtained from this study.
