# Supplementary material for: Integrating a Numerical Taxonomic Method and Molecular Phylogeny for Species Delimitation of Melampsora Species (Melampsoraceae, Pucciniales) on Willows in China
Source: PLoS One. 2015 Dec 17;10(12):e0144883. doi: 10.1371/journal.pone.0144883 (PMC4683050; doi:10.1371/journal.pone.0144883)
Supplement: S3 Table — (DOCX) [file pone.0144883.s003.docx]

| **Scenario** | **-lnL** | **K** | **⊿lnL** | **Bonferroni**  **corrected P** |
| --- | --- | --- | --- | --- |
| **1-species** | **5492.8319** | **2** | **0** |  |
| **12-species** | **5433.7235** | **14** | **188.107** | **0.8** |
| **29-species** | **5165.2841** | **29** | **317.0215** | **P<0.0001** |
